# A review of antimicrobial usage practice in livestock and poultry production and its consequences on human and animal health

**DOI:** 10.5455/javar.2024.k817

**Published:** 2024-09-29

**Authors:** Md. Ariful Islam, Palash Bose, Md. Zaminur Rahman, Muhammad Muktaruzzaman, Papia Sultana, Tanvir Ahamed, Mst. Minara Khatun

**Affiliations:** Department of Microbiology and Hygiene, Bangladesh Agricultural University, Mymensingh, Bangladesh

**Keywords:** Antimicrobial usages, antimicrobial resistance (AMR), livestock, health, hazards poultry

## Abstract

Antimicrobials are employed in the control of contagious illnesses in humans and animals and are also utilized as growth enhancers in livestock and poultry. Improper application of antibiotics results in the development of multi-drug-resistant (MDR) bacteria, such as methicillin-resistant *Staphylococcus aureus *(MRSA), vancomycin-resistant *S. aureus *(VRSA), colistin-resistant, extended-spectrum beta-lactamase (ESBL)-producing *E. coli,* and fluoroquinolone-resistant *Salmonella*. Transmission of MDR bacteria happens among animals, from human to animal, and vice versa, resulting in treatment failure, increased treatment cost, and high morality. In this article, we analyzed the recent publications of the current antimicrobial application practices in livestock and poultry farms and the development of antimicrobial resistant (AMR) bacteria in livestock and poultry and its adverse effects on human and animal health using PubMed, Google Scholar, and Google. Citations from published articles were also analyzed. Several drug-resistant bacteria, including MRSA, VRSA, colistin-resistant strains, ESBL-producing *E. coli*, and fluoroquinolone-resistant *Salmonella*, have emerged due to heavy antibiotic application in cattle and poultry, according to the analysis. Transmission happens between people and animals as well as throughout the production chain, which raises the chance of failure of antibiotic therapy and fatality. To stop the proliferation of drug-resistant bacteria, it is important to ensure the proper use of antibiotics in livestock and poultry. Especially in developing nations, strict control and implementation of antimicrobial rules are necessary. To successfully address antimicrobial resistance and lessen dependency on antibiotics, alternative disease management strategies in livestock and poultry must be developed.

## Introduction

Antibiotics are chemical substances produced by microorganisms that either kill (bactericidal) or prevent (bacteriostatic) the growth of other microorganisms [1]. Antibiotics produced by bacteria are mostly live in soil [2]. Penicillin, the first antibiotic, was discovered in 1941. There are various ways that antibiotics combat microorganisms. It hinders the production of the cell membrane, cell wall, protein, and vital enzymes [3]. Narrow-spectrum and broad-spectrum antibiotics can be broadly divided into two groups. Medication with a narrow spectrum (such as penicillin, streptomycin, and so on) only inhibits the growth of particular types of bacteria, such as Gram-positive or Gram-negative. Tetracycline, chloramphenicol, and other antibiotics with a broad range of action are effective against both Gram-positive and Gram-negative bacteria [4]. Some types of antibiotics and their mechanisms of action are summarized in Table 1.

The increased demand for meat and milk for human consumption leads to a huge expansion of intensive livestock and poultry farms both in rich and poor countries across the globe. Antibiotics are necessary for the therapeutic management of illness in both people and animals and for the improvement of food safety, animal welfare, and production. The most often used antibiotics in livestock and poultry sectors include tetracycline, colistin, ciprofloxacin, tylosin, neomycin, amoxicillin, trimethoprim, sulfonamides, doxycycline, erythromycin, and tiamulin [5]. These medications are frequently used to treat bacterial and other infectious diseases. Antibiotics can also be used to treat certain fungus, chlamydial, and rickettsial diseases [6]. In developing countries, poor biosecurity practices, less vaccination coverage against bacterial diseases, and a lack of adequate nutritious feed are often associated with disease outbreaks in livestock and poultry farms. Antibiotic usage in animals and poultry as a preventative measure is a standard procedure to halt the spread of infectious diseases [7]. To boost livestock and poultry production, antibiotics are often utilized as growth promoters [8,9]. Animals’ guts develop antimicrobial resistant (AMR) bacteria as a result of antibiotic use at accurate or low doses in feed and water. The application of antibiotics as growth enhancers is responsible for the rise of AMR bacteria [10].

**Table 1.** Antibiotic classes and their mode of action.

Class of antibiotics	Name of antibiotics	Mechanism of action	References
Aminoglycosides	Gentamicin, streptomycin, neomycin, and paromomycin.	Inhibit protein synthesis.	[57]
Cephalosporin	Cefoxitin, cephalexin, ceftaroline, cefuroxime, cefroxadine and ceftezole.	Inhibit cell wall synthesis.	[58]
Tetracycline	Tetracycline, oxytetracycline, doxycycline, minocycline and tigecycline.	Inhibit protein synthesis.	[59]
Penicillin	Methicillin, oxacillin, flucloxacillin, ampicillin, amoxicillin and benzyl- penicillin.	Inhibit cell wall synthesis.	[60]
Sulfonamides	Sulfadiazine, sulfafurazole, sulfadoxine and methazolamide.	Inhibit folate synthesis.	[61]
Fluoroquinolones	Ciprofloxacin, norfloxacin, levofloxacin and moxifloxacin.	Inhibit DNA replication.	[62]
Macrolides	Azithromycin, erythromycin, carbomycin a and nystatin.	Inhibit protein synthesis.	[63]
Glycopeptides	Vancomycin, telavancin, corbomycin, bleomycin, lipopeptides and polymyxins.	Inhibit cell wall synthesis.	[64]
Lincosamides	Clindamycin, lincomycin and pirlimycin.	Inhibit protein synthesis.	[65]
Carbapenems	Imipenem, meropenem, doripenem and razupenem.	Inhibit cell wall synthesis.	[66]

Antibiotics are widely available to farmers without a prescription, which contributes to antibiotic overuse. Because of AMR, which has emerged as a result of antibiotic abuse, some infections can no longer be treated [11]. The extensive use and misuse of antimicrobial drugs in livestock farms led to the development and spread of antimicrobial resistance. There have been reports of farmers abusing antibiotics that were available without a veterinarian’s prescription [12].

Extended-spectrum beta-lactamase (ESBL)-producing *E. coli*, Methicillin-resistant *S. aureus *(MRSA), Vancomycin-resistant *S. aureus* (VRSA), colistin-resistant *E. coli*, and fluoroquinolones-resistant *Salmonella* are the most common multidrug resistant (MDR) bacteria in people and animals [13]. Numerous reports demonstrate the spread of MDR pathogens from animal to human and inversely [14]. Consuming foods derived from animals (such as eggs, meat, and milk) that are contaminated with the MDR bacteria as well as coming into direct contact with animals are two ways that humans might become infected with the MDR bacterium [15]. The AMR is a serious public and animal health threat around the world [16]. It is yet unclear how the widespread utilization of antibiotics and the emergence of AMR affect the livestock industry. There are still many gaps in our present knowledge of how AMR develops and spreads in the cattle and poultry industries [17]. This study addresses the present use of antibiotics in livestock and poultry farms, the transmission of AMR bacteria in livestock and poultry, and the health hazards caused by AMR to humans and animals.

## Methodology

A search of the review of the literature was conducted in PubMed, Google Scholar, and Google to meet the objectives. The right combination of keywords was employed, such as antimicrobial usages (in cattle and poultry), antimicrobial resistance (AMR and MDR), and health risks (human and animal). We also looked for and reviewed the articles cited in this review.

## Results and Discussion

### Antimicrobial usage in livestock farming

Antimicrobials are used in animal husbandry for several purposes, such as prophylaxis (preventive treatment), metaphylaxis (therapeutic management of the whole herd whenever a disease epidemic occurs), and growth stimulation [18]. Brazil, Russia, India, China, and South Africa (BRICS) are countries that have turned toward vertically integrated, extremely cost-efficient intensive livestock production systems to meet the massive demand for protein for their people [19]. Antimicrobials are necessary in these production systems to ensure the well-being of animals and productivity. The utilization of antimicrobials in cattle has been rapidly increasing in developing countries in South and Southeast Asia, including Bangladesh, India, Nepal, Bhutan, Sri Lanka, Myanmar, Thailand, and Indonesia [20], as well as in the African countries of Ethiopia and Tanzania [18,21] in 2017. The World Health Organization (WHO) instructed its member nations to minimize the application of antibiotics in food animals. The European Union (EU) has prohibited the utilization of antibiotics for growth enhancement since 2006. In the United States, the application of low concentrations of antibiotics through feed and water to increase the ratio of feed conversion and health was declared illegal in 2017. The application of antibiotics in livestock farms is loosely controlled and regulated in many low-middle-income nations [22]. This results in the improper usage of antibiotics. Farm animals consume 73% of all antimicrobials throughout the world, although the amounts of antibiotic consumption are different among countries. For example, animals are treated with small quantities of antibiotics in Northern European countries when compared to humans. A study conducted in 2015 stated that developing BRIC nations are expected to increase the use of agricultural antibiotics by 67% between 2010 and 2030. The two main factors driving the increase in antimicrobial usage in agriculture are the increased livestock output and consumer demand for animal items [19]. Table 2 displays antimicrobial usage practices by nation for farm animals.

### Antimicrobial usage in poultry farming

In order to prompt growth, prevention, and control of diseases in poultry, antibiotics are frequently used on the entire flock [23]. Countries that produce poultry have the provision for using antibiotics for the prevention of infectious diseases. Intestinal infections such as colibacillosis, necrotic enteritis, and other illnesses are treated with antibiotics [24]. The class and intensity of AU practices vary from country to country depending on the country’s financial status, as well as the degree of development, animal-rearing methods, and animal species [25]. Depending on the stage of production and the danger of disease, different antibiotic dosages and administration techniques are used [26]. Table 3 displays antimicrobial usage patterns for poultry by country.

**Table 2.** Country-wise antimicrobial usage in livestock farming.

Country	Antimicrobial usage practice	References
Ethiopia	The types of antibiotics used in different agro-ecological zones and production systems were reported to be used differently. Antibiotics were most frequently used by pastoralists (86.7%), then anthelmintics (70.8%). Among all the study sites, the most commonly utilized antibiotic classes were trimethoprim-sulfonamides (6.2%), aminoglycosides (31.3%), and tetracyclines (36.4%).	[18]
Tanzania	Penicillin (36.4%) was the most often used antimicrobial agent in the small-scale dairy farms having records of antimicrobial use. Sulfamethoxazole (22.3%), oxytetracycline (14.3%), and dihydrostreptomycin were the next most commonly used antimicrobial agents (11.5%).	[21]
Bangladesh	Antibiotics are mostly used to treat illnesses in livestock. The most commonly prescribed antibiotics were streptomycin–penicillin (31%) followed by sulfadimidine (14%), amoxicillin (11%), gentamicin–sulfadiazine–trimethoprim combination (9%), and tylosin (1%).	[67]
Bhutan	All animals with wounds and diarrhea were regularly treated with broad-spectrum antibiotics such as penicillin, tetracyclines, trimethoprim + sulfa, and sulfonamides. Prescriptions for antibiotics fall under the AWaRe access group (45%–70%) and watch group (up to 25%).	[68]
India	In milk-producing animals, antibiotics are frequently used for both therapeutic and preventative objectives. According to estimates, India used 3% of the world‘s antibiotics used in food animal production in 2010, ranking fourth in the world.	[11,19]
Myanmar	The majority of medications are utilized as feed additives for sub-therapeutic prevention, growth promotion, and therapy. Β-lactams, tetracycline, fluoroquinolones, aminoglycosides, macrolides, and sulphonamides are among the principal antimicrobial classes utilized in Myanmar‘s livestock industry.	[25]
Nepal	More than 70% of veterinary medicine sales were from paraprofessionals or retail establishments. The top seven antibiotics used in Nepal are tetracycline, enrofloxacin, neomycin-doxycycline, levofloxacin, colistin, and tylosin, with ampicillin, amoxicillin, ceftriaxone, and gentamicin being the drugs most frequently administered incorrectly.	[69,70]
Sri Lanka	The most often used antibiotic is tetracycline. Combinations of the antibiotics sulfa trimethoprim, cloxacillin, bacitracin, and neomycin are frequently used to treat mastitis.	[71]
Indonesia	Tetracycline and amoxicillin were the most frequently used antibiotics, being used by 28.7% of the farmers. Benzylpenicillin, gentamicin, ciprofloxacin, oxytetracycline, enrofloxacin, and doxycycline were other antibiotics utilized.	[72]

**Table 3.** Country-wise antimicrobial usage in poultry farming. <AQ3>

Country	Antimicrobial usage practices	References
Nigeria	Tetracycline was the most preferred antimicrobial medicine among farmers (41.0%), while amoxicillin and a combination of oxytetracycline, amoxicillin, colistin, and streptomycin were the lowest ranked (1.0 %).	[73]
Tanzania	Seven classes and seventeen different antimicrobial agents were identified in chicken farms. The majority of farms (87.7% of poultry farms) used antibiotics for medical treatment. The drug withdrawal periods were not followed by about 41% of the chickens. The most often used antimicrobials on these farms included beta-lactams, fluoroquinolones, sulphonamides, tetracyclines, and macrolides.	[21]
Bangladesh	Of the antimicrobials used in layer farms (17/54), ciprofloxacin (37.0%), amoxicillin (33.3%), and tiamulin (31.5%) were the most commonly utilized. The three drugs with the highest usage rates on broiler farms were colistin (56.6%), doxycycline (50.6%), and neomycin (38.6%). 84.7% of farmers used antimicrobials prophylactically, compared to only 15.3% who used them only for therapeutic purposes. Overall, 75.2% of farmers used antibiotics in clinical conditions, while 24.8% of farmers used antibiotics as preventative measures.	[10,74]
Nepal	Six farms (22%) used antibiotics for prophylactic, while 21 (78%) utilized them for therapeutic purposes. Quinolones, macrolides, and polymyxins were among the seven antibiotics administered, which came from six different classes. Tylosin (47%), colistin (47%), and neomycin and doxycycline dual treatments were the most widely utilized antibiotics (33%).	[75]
Vietnam	The three most common antimicrobials in chicken feed formulations were bacitracin (15.5% feeds), 11.4% chlortetracycline, and 10.8% enramycin. Antimicrobials such as amoxicillin, colistin, tetracyclines, neomycin, lincomycin, and bacitracin that the World Health Organization has determined to be relevant for use in human medicine accounted for 57% of the total quantitative consumption.	[76]
China	The most prevalent antimicrobials used in the production of poultry are coccidiostats and arsenicals. There are also concerns about antimicrobial resistance and public health due to the use of macrolides, penicillins, and tetracyclines.	[77]
Columbia	In chicken farms, antibiotics are frequently used as feed growth enhancers and as a treatment for the most prevalent infectious diseases, like bronchitis and infected bursal disease. There were numerous reports of the usage of antimycoplasma agents (65.7%). Overall, calcium phosphomycin (44.3%), enrofloxacin (44.3%), ciprofloxacin (31.4%), norfloxacin (12.9%), and trimetho-prim-sulfamethoxazole were the most often reported antimicrobials utilized for treatments (18.6%).	[78]

### Antimicrobial-resistant bacteria transmission pathways originating from livestock and poultry

Animals can transmit their resistance to humans in several ways, the food-borne route is the most important. Other ways of transmission consist of coming into direct contact with animals, eating contaminated foods, and indirectly through a polluted environment [27,28].

In developed nations, the food-borne route is likely the pathway by which the majority of infections with enteric bacterial pathogens, such as *Salmonella entererica*, *Campylobacter coli/jejuni*, and *Yersinia enterocolitica,* occur. Humans can contract MRSA CC398 through direct animal contact, environmental contamination, and handling or consuming contaminated meat [29]. Table 4 shows the transmission pathways of AMR bacteria originating from poultry.

### Prevalence of MDR bacteria in livestock and poultry

Factors that help develop antibiotic resistance (AMR) include bacterial mutation, spontaneous evolution, and horizontal gene transfer [30]. Through bacterial evolution and mutation, AMR could arise naturally [31]. Furthermore, transposons and insertion sequences allow plasmids—small circular DNA fragments that are common in bacteria—to acquire a wide range of resistance genes [32]. Antibacterial resistance among different species of bacteria can be spread by plasmid [33]. Furthermore, the transmission of AMR is sped up by bacteria harboring resistance genes with one another by horizontal gene transfer [34].

**Table 4.** Country-wise antimicrobial resistance transmission pathways originating from poultry.

Country	Location	AMR transmission (s) pathways	Operation level	Key findings	References
India	Urban	Intensive farming	Large	*E. coli* with a high rate (94%) of ESBL production and multidrug resistance (87%).	[79]
Zimbabwe	Peri-urban Countryside Urban	Intensive farming	Small Large	The intensity of farming is correlated with higher AMR levels of *Salmonella *spp. Public health is at risk from salmonellosis when isolates of *S. enteritidis *are MDR in 12.1% of cases.	[80]
Kenya	Countryside	Intensive farming	Small Large	Thermophilic Campylobacter species with confirmed antibiotic resistance in small-scale chicken systems.	[81]
Nigeria	Urban	AMR transmission across species	Large	Samples of chicken and cattle excrement were discovered to have high amounts of AMR and virulent *Enterococcus *spp., suggesting cross-species transmission.	[82]
Ecuador	Countryside	AMR transmission across breeds Transmission of Zoonotic AMR	Small	The amount of *E. coli* that produces the beta-lactamase CTX-M in backyard hens increased significantly (66.1%). The sequencing of blaCTX-Mrevealed a high degree of similarity between human, broiler chicken, and backyard chicken samples from the villages, which may point to zoonotic transmission of AMR.	[83]
India	Countryside	Transfer indirectly to backyard chickens	Small	MDR and avian pathogenic *E. coli* related virulence genes were found in high prevalence in the backyard layer chickens and their environment 75.5%. Possibility of AMR contamination in surrounding ponds and/or flocks of commercial broiler chickens due to human feces.	[84]
Ecuador	Countryside	Transfer indirectly to backyard chickens	Small	There have been reports of thermophilic resistant Campylobacter species in untreated backyard hens given unrestricted freedom to roam.	[85]
`Bangladesh	Urban	Intensive farming Zoonotic	Large Medium	MDR* Escherichia coli *isolated from hands of poultry workers, intensive poultry operations, and poultry husbandry environments.	[86]
Costa Rica	Countryside	Spread to wild avian specie	Small	Neotropical birds are at risk of contracting resistant *E. coli* from free-ranging chickens.	[87]
Kenya	Countryside	Transfer indirectly to backyard chickens	Small	Salmonella spp. and E. coli that were isolated from backyard chicken feces were found to have class 1 integrons beta-lactamase genes.	[88]
Vietnam	Countryside	Intensive farming Exposure at Work	Small Medium	Showed a connection between farmers and intensively farmed poultry and AMR *Salmonella *spp.	[81]

MDR: Multidrug-resistant, AMR: Antimicrobial resistant.

Several factors are accelerating the rate of AMR bacteria: (i) Antibiotic prescriptions are the only means of providing patient treatment in many countries without sufficient medical diagnostic skills. This might result in the improper or excessive administration of antibiotics; (ii) self-treatment of antibiotics is often practiced in countries where antibiotics are available without a prescription; (iii) treatment of disease conditions with antibiotics where these are not required; and (iv) administration of antibiotics to healthy animals’ food and water to promote growth [35]. Table 5 lists the prevalence of MDR bacteria by country in livestock and poultry.

### Consequences of antimicrobial usage in human and animal health

**Reduced effectiveness of antibiotics**

The misuse of antibiotics in public health encourages the growth of bacteria resistant to current drugs and lessens their efficacy [36]. Individuals suffering from chronic medical conditions such as diabetes, asthma, and rheumatoid arthritis are particularly vulnerable to the impact of AMR [30]. Doctors could have to employ antibiotics from the last-resort classes, such as carbapenems and polymyxins. These medications are costly, not always easily accessible in impoverished nations, and have several negative effects. The persistence trends of AMR reduce the effectiveness of antibiotics [30].

**Table 5.** Country-wise prevalence of MDR in livestock and poultry.

Country	Specimen types	MDR Bacteria	Prevalence (%)	References
Bangladesh	Poultry	*E. coli*	36.6	[89]
	Milk	*C. jejuni*	57.1	[90]
		*C. coli*	33.33	
	Poultry	*C. jejuni*	49	[91]
		*C. coli*	42	
	Milk	*Salmonella *spp.	100	[92]
	Beef	*Salmonella *spp.	66.67	
	Poultry meat	*Salmonella *spp.	93.10	
	Milk	*S. aureus*	12	[92]
	Poultry meat	*S. aureus*	53.85	
	Egg	*S. aureus*	90.91	
Bhutan	Pig fecal sample	*E. coli*	2.4	[93]
India	Bovine	*E. coli*	71.43	[94]
	Poultry	*E. coli*	63.2	[95]
	Piglets	*E. coli*	80	[96]
	Bovine	Shiga toxin-producing *E. coli*	17	[97]
	Poultry	*Salmonella *spp.	100	[84]
	Bovine	*S. aureus*	20–30	[98]
Indonesia	Poultry	*E. faecalis*	84.5	[99]
Myanmar	Poultry	*Salmonella *spp.	52.2	[100]
Nepal	Buffalo	*E. coli*	52.5	[101]
	poultry meat	*Proteus *spp.	77.7	
		*S. aureus*	40.0	
Sri-Lanka	Pigs, chickens, and cattle	*S. aureus*	65	[102]
Thailand	Poultry	ESBL-producing *S. Typhimurium*	77.3	[103]
	Pig	ESBL-producing *S. Typhimurium*	40.4	
	Pig	ESBL-producing *E. coli*	77	[104]
	Pork	ESBL-producing *E. coli*	61	
	Pork	*A. baumannii *and *P. aeruginosa*	40	
	Poultry	ESBL-producing *E. coli*	40	
	Poultry meat	ESBL-producing *E. coli*	50	

MDR = Multidrug resistant, ESBL = Extended spectrum beta lactamase.

**Treatment failure**

AMR may result in early treatment failure because it delays the start of effective antibacterial medication and forces doctors to hunt for more toxic and rare antibiotic alternatives. Gram-negative bacteria that are resistant to drugs multidrug resistant-gram negative bacteria (MDR-GNB) have made treating several illnesses, including pneumonia and urinary tract infections, more difficult [37]. Two types of bacteria that are generally resistant to most commercially available antibiotics include vancomycin-resistant enterococci and methicillin-resistant *Staphylococcus aureus* [38].

**Increased mortality**

Infections resistant to antibiotics are becoming a more serious medical and public health issue in the US. MDR, in particular, is an AMR in bacteria that poses a worldwide health risk to humans and animals [33]. According to estimates from the Centers for Disease Control and Prevention (CDC), infections caused by AMR claim the lives of 23,000 people annually [39]. According to predictions, MDR will cause 10 million more fatalities in humans by 2050 than cancer will [40]. MRSA, is one of the most important antimicrobial resistance (AMR) bacteria, associated with increasing annual death rates globally [41].

**Increase treatment costs**

Antibiotic-resistant illnesses place a heavy cost on a nation’s healthcare system. When first- and second-line antibiotic therapy options are few or nonexistent, healthcare professionals may be forced to use antibiotics that are costlier and damaging to the patient [42]. According to Prestinaci et al. [12] and Shrestha et al. [43], AMR contributes to treatment failures, increased expenses for second-line treatments, lengthier hospital admissions, and catastrophic illnesses. The EU is estimated to lose more than 9 billion euros a year as a result of AMR [12,44]. Drug resistance to typhoid fever, gonorrhea, and tuberculosis is growing annually and significantly contributes to the high costs of healthcare for individuals and systems globally, especially in developing nations [44].

**Direct transfer of MDR bacteria**

Individuals working in farms and slaughterhouses, veterinarians, and farm laborers themselves are directly exposed to the danger of acquiring resistant bacteria through close contact with animals that are colonized or diseased [45]. Tetracycline- and penicillin-resistant *E. coli* was discovered to have infected poultry farm workers and family members through direct or indirect contact with poultry [46,47]. Swine farmers were known to be infected with drug-resistant *S. aureus*, *Streptococcus* spp., and *E. coli *[48]. MDR bacteria MRSA ST398 were transferred to Dutch veal farmers from Dutch veal calves [49]. For years, reports indicated that the increased frequency of bacteria resistant to several drugs might be attributed to the misuse of antibiotics in veterinary treatment or as growth promoters in animal feed [50].

Salmonella’s selection for the plasmid-carried gene *aac* (3)-IV suggests that using apramycin in production animals causes an increase in the development of resistance to aminoglycosides, such as gentamicin and netilmicin [51]. *Enterococcus* that is resistant to high-level aminoglycoside resistance, vancomycin-resistant, and MDR *Enterococcus* pose specific concerns [52]. Spanish chickens that had been slaughtered were the source of the antibiotic-resistant *E. coli* that hospital patients were confirmed to be afflicted with [53].

**Transfer of drug-resistant genes**

There is speculative evidence that bacteria can transfer resistance genes, like the *vanA* gene cluster or genes that give new generations of cephalosporins resistance, to both humans and animals [54]. The resistance-specific blaCMY gene was present in all resistant *Salmonella* *enterica* serotype Newport isolates from humans, pigs, cattle, and poultry [44].

**Antibiotic residues**

The production of food animals, mostly through manure, leaves behind a large number of bacteria and antibiotic residues in the environment that impact both natural bacteria and wild animals. Because of this, reservoirs for food-animal and human consumption may be refilled with resistant bacteria from the environment and wild animals [29]. Public health appears to be less at risk from antimicrobial residues that may be present in items generated from animals than it is from germs that are resistant to them. According to a 2003 report by a WHO expert committee, foodborne pathogens resistant to antibiotics and the accompanying risks are far greater than the risk associated with antimicrobial residues in food under the current regulatory frameworks [55]. Foods originating from animals may contain antimicrobial residues that might lead to serious health problems in humans, including allergic responses, AMR, mutagenesis, and carcinogenesis [56].

## Conclusion

Regarding human health, AMR is a serious concern. Antimicrobial drugs are indiscriminately used in the production of livestock and poultry around the world for treatment, disease prevention, and growth promotion. Animals’ gut-developing AMR bacteria are linked to using antimicrobials at sub-therapeutic doses for longer periods. AMR bacteria from animal guts can contaminate food, water, and the environment. These AMR bacteria are spread via contaminated food, water, and soil to both animals and humans. By coming into direct contact with animals, humans can contract the AMR bacteria. Animals given antibiotics keep residues of the drugs in their tissues, and humans can contract them by consuming milk, meat, or eggs. Through horizontal gene transfer methods, drug-resistance genes are transferred from one bacterium to another. Human and animal health risks from the AMR bacteria include treatment failure, an increase in mortality, and an increase in treatment costs. To combat the rise of AMR, prudent antimicrobial agent use in livestock and poultry production is crucial. One way to limit the spread of antibiotic-resistant bacteria in poultry and animals is to offer them probiotics and prebiotics. Other strategies include adapting biosecurity measures in livestock and poultry farms and vaccinating against infectious diseases.

## List of Abbreviations

AMR, Antimicrobial Resistant; EU, European Union; ESBL, Extended Spectrum Beta-Lactamase; MDR, Multidrug Resistant; MRSA, Methicillin Resistant *Staphylococcus aureus*; VRSA, Vancomycin Resistant *Staphylococcus aureus*; WHO, World Health Organization.
